# Transcriptome analysis identified a novel 3-LncRNA regulatory network of transthyretin attenuating glucose induced hRECs dysfunction in diabetic retinopathy

**DOI:** 10.1186/s12920-019-0596-2

**Published:** 2019-10-15

**Authors:** Jun Shao, Yunbin Zhang, Guangming Fan, Yu Xin, Yong Yao

**Affiliations:** 1 Department of Ophthalmology, Wuxi People’s Hospital affiliated to Nanjing Medical University, Wuxi, 214023 Jiangsu China; 2 Institute of Biochemistry and Cell Biology, Chinese Academy of Sciences, Shanghai, 200031 China; 3 Key Laboratory of Industry Biotechnology, Ministry of Education, School of Biotechnology, Jiangnan University, Wuxi, 214122 Jiangsu China

**Keywords:** Diabetic retinopathy, Transthyretin, Transcriptome, 3-lncRNA regulatory network, diagnosis

## Abstract

**Background:**

Diabetic retinopathy (DR) is the leading cause of blindness in the working age population. Transthyretin (TTR) showed a significantly decreased concentration in DR patients and exerted a visual protective effect by repressing neovascularization. This work intended to identify long non coding RNAs (lncRNAs) and explore their potential mechanism underlying the protective role of TTR.

**Methods:**

Transcriptome of human retinal endothelial cells (hRECs) treated with low glucose (LG), high glucose (HG) or high glucose with 4 μM TTR (HG + TTR) was conducted. Differentially expressed lncRNAs, mRNAs and TTR related lncRNAs and mRNA were acquired. Functional annotation and Gene Set Enrichment Analysis were applied to analyse TTR affected pathways and processes. Weighted gene co-expression network analysis (WGCNA) was implemented to obtain hub modules and genes. LncRNA-mRNA regulatory networks were constructed based on *cis*, *trans* and competing endogenous RNAs acting mode. QRT-PCR was conducted to validate the expression of lncRNAs in aqueous humor and serum samples from 30 DR patients and 10 normal controls.

**Results:**

RNA-sequencing of hRECs treated with low glucose (LG), high glucose (HG) or high glucose with 4 μM TTR (HG + TTR) was conducted. 146,783 protein-coding transcripts, 12,403 known lncRNA transcripts and 1184 novel non-coding transcripts were characterized. A total of 11,407 differentially expressed mRNAs (DE-mRNAs), 679 differentially expressed lncRNAs (DE-lncRNAs) in HG group versus LG group, 6206 DE-mRNAs and 194 DE-lncRNAs in HG + TTR versus HG group were obtained, respectively. 853 TTR-mRNAs and 48 TTR-lncRNAs were acquired, and functionally involved in cell cycle, apoptosis, inflammation signalling pathway, response to oxidative stress, neovascularization and autophagy. The WGCNA analysis identified a hub module of 133 genes, with the core function of oxidative stress response, angiogenesis, MAPK pathway, cell proliferation and apoptosis. After qRT-PCR validation, a 3-lncRNA regulatory network was proposed. At last, lncRNAs *MSTRG.15047.3* and *AC008403.3* showed significantly relative higher expression levels in both aqueous humor and serum samples, compared with normal controls, and *FRMD6-AS2* was significantly down-regulated.

**Conclusions:**

TTR regulated mRNAs and biological processes including oxidative stress, inflammation signalling and autophagy. A 3-lncRNA regulatory network was characterized underlying TTR repressing neovascularization, and showed potential diagnostic performance in DR.

## Background

As the leading cause of vision impairment and loss in the working age population, diabetic retinopathy (DR) is a common and specific microvascular complication characterized by early retinal microvascular dysfunction [1]. In China, the number of DR patients is growing each year [2]. The early stage of DR is exemplified by retina endothelial cells and pericytes death [3]. As DR progresses, vascular leakage becomes evident and may eventually lead to diabetic macular edema, the most common cause of vision loss in DR [4]. Till now, panretinal photocoagulation, vitrectomy and anti-neovascularization drug intraocular injection remain the few therapeutic options [5]. Accumulating evidence implicated that inflammation, cell apoptosis, oxidative stress and autophagy were major causative factors involved in the pathogenesis of DR [6]. Therefore, uncovering potential mechanisms underlying DR pathogenesis, especially revealing hub genes and pathways in inflammation, cell apoptosis, oxidative stress and autophagy is of paramount importance for the development of effective therapeutics for patients with DR.

Transthyretin (TTR) is a 55-kDa homotetramer protein reported to transport thyroxine and retinol through retinol-binding protein (RBP) [7], and it is mainly secreted by human retinal pigment epithelial cells (hRPECs) and choroid in ocular tissue. In vitreous and serum samples of high myopia patients, TTR showed significantly higher expression compared with healthy donors [8]. Interestingly, the expression of TTR in DR patients was significantly lower, compared with healthy donors [8, 9]. In our previous research, an exogenous 4 μM TTR treatment or endogenous TTR overexpression both repressed blood vessel tube forming capability of hyperglycemic cultured human retinal endothelial cells (hRECs), by attenuating pro-angiogenic genes expression, like Angiopoietin 2 (*Angpt2*), Vascular Endothelial Growth Factor Receptor 1 (*VEGFR1*), and Vascular Endothelial Growth Factor (*VEGF2*) [10, 11]. Additionally, TTR was reported to repress neovascularization by promoting hRECs apoptosis through direct binding to glucose-regulated protein 78 (*GRP78*) [12]. Surprisingly, the exogenous added TTR efficiently crossed cell membrane and nuclear membrane, and located in both in cytoplasm and nucleus, implicating complex transcriptional and post-transcriptional regulatory role, while the exact mechanism still remains obscure.

Long noncoding RNA (lncRNA) is characterized to be transcripts with a length of more than 200 nt and of no protein-coding capability. Accumulating evidence pointed that lncRNA exerted function in *cis* by regulating nearby genes, or in *trans* by modulating distantly located genes [13]. In addition, it’s a putative mechanism that lncRNAs may regulate target gene expression via miRNA response element (MRE), known as competing endogenous RNA (ceRNA) [14]. The aberrant expression of lncRNAs in early diabetic retinopathy was first characterized in 2004 [15]. Subsequent studies of individual lncRNAs playing a transcriptional or post-transcriptional regulatory role in DR pathogenesis and progression had been reported, including Maternally Expressed 3 (*MEG3*) [16], Myocardial Infarction Associated Transcript (*MIAT*) [17, 18], HOXA Distal Transcript Antisense RNA (*HOTTIP*) [19], Metastasis Associated Lung Adenocarcinoma Transcript 1(*MALAT1*) [20, 21], SOX2 Overlapping Transcript (*Sox2-OT*) [22], antisense non-coding RNA in the INK4 locus (*ANRIL*) [23] and BDNF Antisense RNA (*BDNF-AS*) [24]. However, there have been limited studies of lncRNAs in TTR repressing DR neovascularization.

In this study, we proposed that there may exist certain lncRNAs, through which TTR exerts its function of repressing neovascularization in DR. RNA transcriptome of hRECs treated with low glucose concentration of 5.5 mM (LG), high glucose concentration of 25 mM (HG), and a 25 mM high glucose plus a concentration of 4 μM TTR (HG + TTR) was determined for the first time. The impact of high glucose and TTR on hRECs neovascularization was evaluated with tube formation assay. Then differentially expressed lncRNAs and mRNAs that induced by high glucose and TTR were identified, respectively. Functional annotation and network analysis of TTR regulated mRNAs significantly enriched pathways and biological processes in DR. Furthermore, the TTR regulated lncRNAs and mRNAs were predicted, and the lncRNA-mRNA regulatory network in *cis*, *trans* and ceRNA was constructed. Finally, after qRT-PCR validation in hRECs, vitreous humor and serum samples, a 3-lncRNA centered TTR response coding-non-coding network with potential diagnosis performance was proposed to explain the protective role of TTR, which would provide basis for the future potential application of TTR and lncRNAs in DR diagnosis and treatment.

## Methods

### Cell culture and TTR treatment

HRECs from donated human retinal tissue were extracted, sub-cultured, and authenticated by Genetic Testing Biotechnology (Shanghai, China) using Short Tandem Repeat (STR) analysis as described in 2012 in ANSI Standard (ASN-0002) by the ATCC Standards Development Organization (SDO). Nineteen short tandem repeat (STR) loci plus the gender determining locus, Amelogenin, were amplified using the commercially available EX20 Kit from AGCU. The cell line sample was processed using the ABI Prism 3130 XL Genetic Analyzer. Data were analyzed using GeneMapper ID v3.2 software (Applied Biosystems). Appropriate positive and negative controls were run and confirmed for each sample submitted. And as detected, eight core STR loci plus Amelogenin revealed that HRECs should be quite similar with HBMEC-2 (human brain microvascular endothelial cell) (Additional files 1, 2 and 3).

HRECs from passage 4 were diluted to 8 × 10^4^/mL and incubated in 6-well plates with low glucose (LG) media until confluent. After washing with PBS, the cells were further cultured for 48 h at 37 °C in 5.5 mM glucose (low group, LG), 25 mM glucose (high glucose, HG), or 25 mM glucose with 4 μM TTR (HG + TTR) media. The concentration of low glucose of 5.5 mM and simulated high glucose concentration of 25 mM were referred to previous studies [25, 26]. The concentration of TTR of 4 μM were referred to Raquel J. Nunes et al’s study; the TTR concentration of 1, 2, 4, 8 and 10 μM were examined and 4 μM was determined as the final concentration [27]. Cells were collected and total RNAs were extracted from hRECs, using TRIzol reagent (Takara, Dalian, China), according to the manufacturer’s instructions.

### Tube formation assay

The basement membrane matrix (BD Biosciences) was placed into the well of 24-well plate, and hardened at 37 °C for 30 min; 2 × 10^4^ hRECs cells were seeded on each well, and incubated with glucose and/ or TTR at 37 °C for 24 h. Tube formation was observed using an Olympus IX-73 microscope.

### Subjects

Aqueous humor and serum samples of 30 DR patients and 10 normal controls (patients without diabetes) were obtained from ophthalmology inpatients of Nanjing Medical University Affiliated Wuxi People’s Hospital. Aqueous humor of normal controls (patients without diabetes) and DR patients were collected during cataract surgery. Clinic patients’ samples processing were the same as we previously reported [9]. Informed consents were signed for enrolled patients and healthy individuals. This study was compiled with principles of the Declaration of Helsinki and was approved by the Ethics Committee of Nanjing Medical University (2014–62). All clinical samples were obtained from ophthalmology inpatients of Nanjing Medical University Affiliated Wuxi people’s Hospital. Aqueous humor sample of DR patients and normal control were obtained from cataract surgery. All samples were stored at − 80 °C.

### Transcriptome sequencing and differentially expressed genes (DEGs) identification

After total RNAs were extracted and collected, cDNA was produced and purified with AMPure XP (Beckman Coulter, Brea, CA, USA) beads. Then PCR was conducted to generate cDNA libraries, and adaptor sequences were removed, followed by low-quality reads filtering. Trinity (http://trinityrnaseq.sourceforge.net/) software and De Bruijn graph algorithm [28] were applied for de novo assembly. At last, unigenes were annotated with BLASTX and BLAST algorithm, by searching GenBank database (http://www.ncbi.nlm.nih.gov/) and NCBI non-redundant (NR) (ftp://ftp.ncbi.nih.gov/blast/db/), respectively. The RNA-seq data had been submitted to GEO repository, with accession number GSE117238.

In this study, we used FPKM (Fragments Per Kilobase of transcript per Million) for transcripts, instead of gene expression, and gene names corresponding to certain transcripts were also shown. For a certain gene, if more than one transcript showed significant differential expression, the gene were considered to be significantly deregulated. Specifically, Student’s t-test and fold change (FC) filtering were conducted, and to screen differentially expressed genes (DEGs) between two groups by using R software limma package. In addition to *P* value, a Benjamini–Hochberg procedure was conducted for multiple comparisons to control the false discovering rate (FDR) [29]. Significantly deregulated transcripts were defined using the criteria of Fold Change≥2, *P* ≤ 0.05 and FDR ≤0.05. Volcano plot were construed by R software ggplot2 package, to show significantly deregulated transcripts and genes. The differential expression analysis was conducted in the transcript level, and transcripts with the converse expression fold-change trend in HG + TTR group versus HG group and HG group versus LG group were named as TTR related DEGs (TTR-DEGs).

### Pathway and GO annotation and GSEA analysis

For differentially expressed genes functional annotation, KEGG pathway and Gene Ontology (GO) annotation and enrichment were conducted, based on Kyoto Encyclopedia of Genes and Genomes (KEGG) (http://www.genome.jp/kegg/) and Gene Ontology database (http://www.geneontology.org/), with DAVID (The Database for Annotation, Visualization and Integrated Discovery v6.8, https://david.ncifcrf.gov/). For GO analysis, biological processes (BP), cellular components (CC) and molecular function (MF) were applied and enriched items were shown, respectively. A Fisher exact test was used to select only significant categories. A Benjamini–Hochberg procedure was conducted for multiple comparisons to control the false discovering rate (FDR) [29]. GO terms with corrected Q value < 0.05 were considered significant. Significantly enriched pathways and biological processes were displayed with R software ggplot2 package.

Compared with functional annotation of DEGs with KEGG pathway and GO, function of all detected genes were analyzed with Gene Set Enrichment Analysis (GSEA) [30]. Specifically, annotation file of c2: curated gene sets-all canonical pathways, gene symbols were used to analyze significantly enriched gene sets. The GSEA analysis was applied to extract significant biological processes and pathways, for HG and TTR + HG groups. Gene expression data matrix of detected genes in LG, HG and TTR + HG groups were imported into GSEA software (v2.2.4) and the permutation index were set as 1000. Then the GS (gene set) score and *P* value were used, and biological processes or pathways with *P* < 0.05 were considered to be significant.

### Protein-protein interaction (PPI) network analysis and sub-network construction

To explore the interaction of TTR-mRNAs, TTR-mRNAs lists were first imported into STRING database (https://string-db.org), and generated the protein-protein interaction (PPI) network. When constructing the PPI network, protein-protein interaction pairs with medium confidence of interaction score > 0.4 were exported and then visualized by using Cytoscape (version 3.4.0). To analyze the topological structure and relationship in PPI network, CentiScaPe was further applied for calculating centrality parameters for each node and finding the most important nodes in a network [31]. At last, Molecular COmplex Detection (MCODE) (http://apps.cytoscape.org/apps/mcode) was applied to find densely connected regions and sub-networks within the main network.

### Weighted gene co-expression network analysis (WGCNA) and module detection

The Weighted gene co-expression network analysis (WGCNA) is a well-known technique used to identify biologically meaningful and functional co-expression modules related to diseases or conditions [32]. In this study, to explore and screen gene expression modules correlated with high glucose and TTR, the gene co-expression network was constructed by the R WGCNA package. The gene expression similarity and soft-threshold were calculated using the integrated function (pick Soft Threshold) in the package. When the soft threshold was defined as 10, a module with ME green color of 133 genes showed highest correlation with both glucose and TTR and was functionally annotated in subsequent steps.

### Module genes biological pathway and function network in ClueGo

The function of 133 ME green module genes were annotated and significantly enriched (*P* < 0.05) pathways were labeled in Cytoscape ClueGo and CluePedia. The ClueGo and CluePedia application were developed to create and visualize a functionally grouped network of terms/pathways [33].

### LncRNA *cis* and *trans* regulatory network construction

For lncRNA targets identification, *cis* and *trans* regulatory network was constructed. To identify *cis* regulatory relationship, lncRNAs that act on neighboring target genes were investigated. We searched for coding genes 10 kb/100 kb upstream and downstream of each lncRNA. To identify *trans* regulatory relationship, lncRNAs and target genes were identified based on their expression levels. We calculated the expression level correlation between lncRNAs and coding genes using custom scripts., and lncRNA-mRNA pairs with correlation coefficient > 0.95 or < − 0.95 were extracted for subsequent analysis. At last, the *cis* or *trans* regulatory network was shown with Cytoscape.

### Competing endogenous RNAs (ceRNAs) network analysis

To reveal the roles and interactions of lncRNAs with mRNAs in glucose and TTR treated hRECs, we constructed lncRNA-miRNA-mRNA regulatory ceRNA networks. Potential miRNA response elements were searched for the sequences of lncRNAs and mRNAs, and we identified overlaps in predicted miRNA seed sequence binding sites as well as lncRNAs binding sites in the target mRNA as part of the lncRNA-miRNA-mRNA interaction. The miRNA binding sites were predicted by miRanda (http://www.microrna.org/), while the miRNA-mRNA interactions were predicted by Targetscan (http://www.targetscan.org/). The interaction network was built and displayed using Cytoscape software based on the screening of lncRNA-miRNA-mRNA pairs.

### QRT-PCR

Cells were collected and total RNAs were extracted from hRECs cells and clinical samples using TRIzol reagent (Takara, Dalian, China), according to the manufacturer’s instructions. Aqueous humor and serum samples were centrifuged at (12,000 g for 10 min at 4 °C) and pellets were collected for RNA extraction and purification. Briefly, cDNA was synthesized by M-MLV reverse transcriptase (Invitrogen, Carlsbad, CA, USA) from extracted RNAs. RT-PCR was performed in triplicate with SYBR Green Real-Time PCR Master Mixes (Takara) on an ABI 7900 PCR system (Applied Biosystems, Bedford, CA, USA). As LncRNA *AC008403.3* was co-localized with *LMTK3* in chromosome 19 and *FRMD6-AS2* and *FRMD6* were localized in DNA+/− strands, with partial sequences overlapping (Additional file 2: Figure S1), transcript specific primers were designed. As previous study pointed that, expression of glyceraldehyde-3-phosphate dehydrogenase (GAPDH), one of the widely used house-keeping genes, was involved in high glucose induced retinal pericytes apoptosis [34], we chose Beta-Actin (*ACTB*) as the house-keeping gene in this study. Information about the primers was listed in Table 1. The RT-PCR program comprised an initial denaturing step at 95 °C for 5 min, followed by 45 cycles of 95 °C for 20 s, 60 °C for 20s, and 70 °C for 20 s. Relative quantification was performed according to the comparative method (2^-ΔΔCT^; Applied Biosystems User Bulletin 2P/N 4303859).

**Table 1:** Primers of genes

Gene	Primer (5′-3′)
FRMD6-AS2	F: CCTGGAACATTGGAAATAGGC R: TGGCAGCATTAGAATACAGCA
AC105345.1	F: TTTGCAGCCTCCTCATCC R: AAAGCCTGTTTGGTGGTCTC
FP236240.2	F: AGATGTCAACACCGCATTAGAG R: CCTGCTTTGCCACTTCCTG
AL392172.1	F: TTGTCCTGGATGCCCTCTT R: CAGACTAGCCAGTCAGTTCTGC
MSTRG.11733.1	F: CCACAGCGATGGCAGATAC R: GAACCACGGAGCGCAAAT
AC087269.1	F: GGACTGGCAAAAGGAGAAAA R: CTGGAGCCAAAATGAAGCA
MSTRG.15047.3	F: AAAAGCACCCGTAGTAGCAAA R: CCAAACTAAAGTAGCCAGCAAG
AL023581.2	F: CCCAACCAATCTGCCTCC R: CCTCCGATTCACTTCTGTTCTTA
AC015813.3	F: CTCCCTCCTCCCGTATCTG R: TTTGCTTCGGCTCTGTCTC
AC008403.3	F: AGAGGGCGGTTGTGAGGA R: ATTTAGTCTGTGCCAGGTATCG
ACTB	F: CGTGGACATCCGCAAAGA R: GAAGGTGGACAGCGAGGC

### Statistical analysis

SPSS 13.0 for windows (Chicago, IL, USA) was used for data analysis, and statistical significance was determined using a T test. The *P* and *T* values were calculated. *P* < 0.05 was considered statistically significant.

## Results

### Exogenous TTR treatment repressed high glucose induced hRECs tube formation

**Fig. 1:**
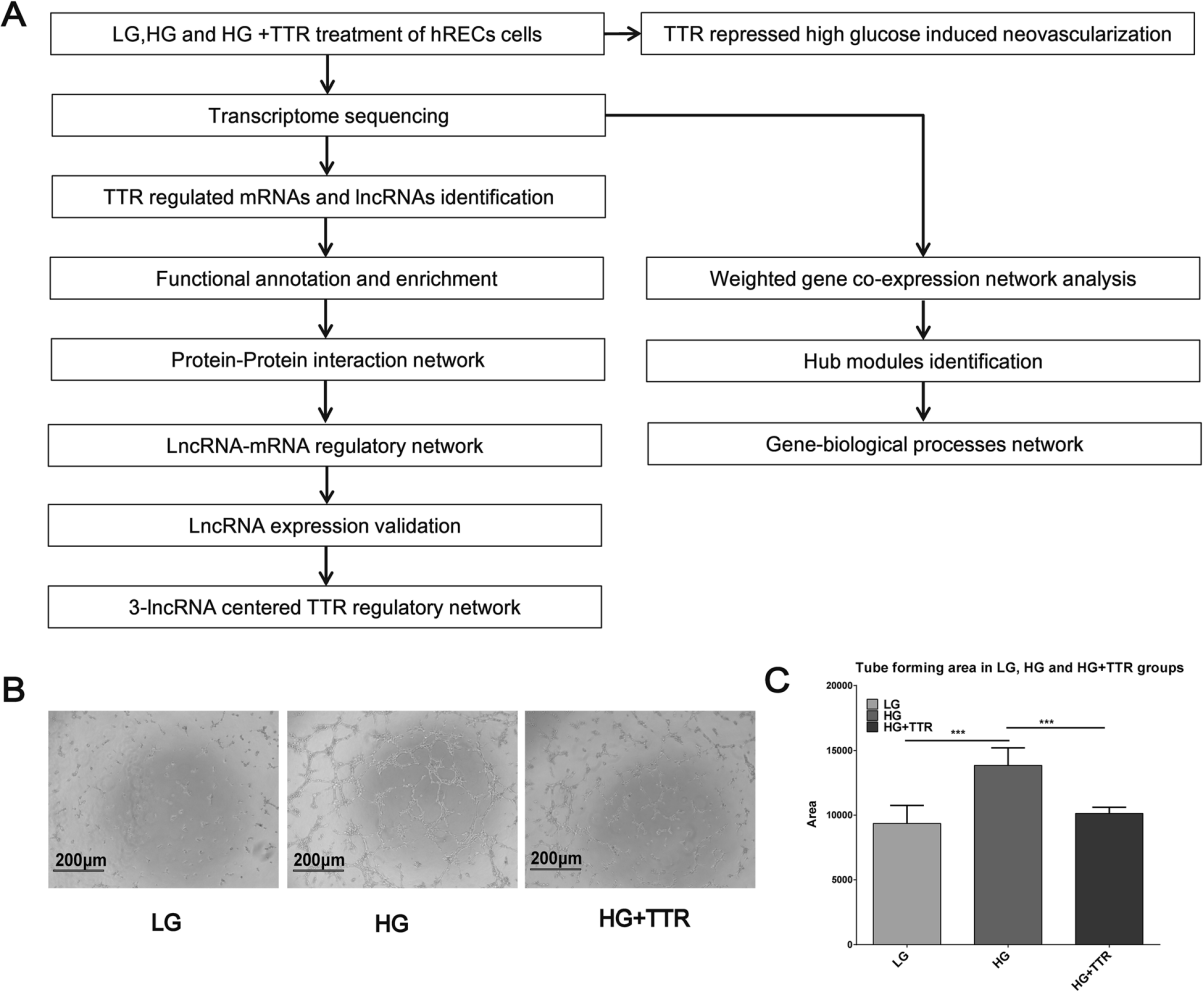
Exogenous TTR treatment repressed high glucose induced hRECs tube formation. **a** Experiment and analysis workflow of this study. **b** In blood vessel tube formation assay, high glucose induced the hRECs neovascularization, and TTR attenuated the effect. **c** Statistics of tube formation area in LG, HG and HG + TTR groups. * *P* < 0.05, ** *P* < 0.01, *** *P* < 0.001

Human retinal endothelial cells (hRECs) were cultured in 5.5 mM glucose (low group, LG), 25 mM glucose (high glucose, HG), or 25 mM glucose with 4 μM TTR (HG + TTR) media. HRECs cellular morphology and physiological nature didn’t vary under different treatment conditions as the treatment is for a long time of 48 h (Additional file 3: Figure S2). Matrigel tube formation assay was conducted to examine TTR impact on the neovascularization. The experiment and analysis workflow was shown in Fig. 1a. Regarding Fig. 1b and c, the tube formation area of HG groups was significantly larger, compared with LG groups (HG average area = 13,843.2, LG average area = 9347.6, HG/LG = 1.48, *P* < 0.001). In HG + TTR co-treatment group, the tube formation area was significantly smaller than HG group (HG + TTR average area = 10,121.2, HG average area = 13,843.2, HG + TTR/HG = 0.73, *P* < 0.001), implicating TTR represses hRECs tube formation in a high glucose environment.

### Transcriptome sequencing characteristics of genes in glucose and TTR treated hRECs

**Fig. 2:**
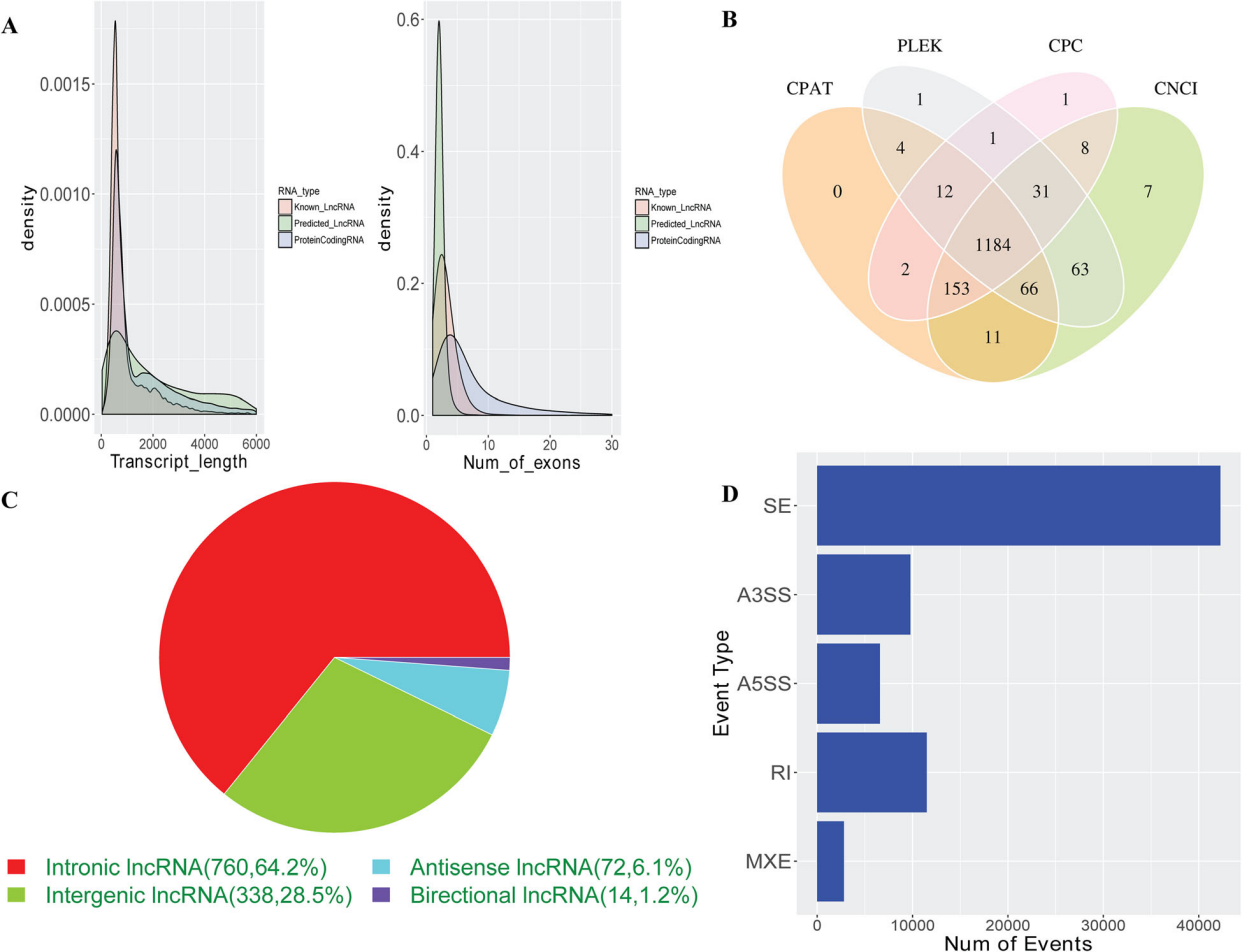
Transcriptome sequencing of genes in glucose and TTR treated hRECs. **a** Distribution of transcripts length and number of exons; **b** Four bioinformatics tools were used to predict novel non-coding transcripts and 1184 novel lncRNAs were identified. **c** Novel lncRNAs categorization based on lncRNA-mRNA location; **d** Alternative splicing events and type

The RNA sequencing of each 3 replicate samples in LG, HG and HG + TTR groups identified a total of 566.19 M unique mapped reads with an average length of 139.4 bp. In total, we characterized 12,403 known lncRNA transcripts and 146,783 protein-coding transcripts. The transcript length and exon distribution were shown in Fig. 2a. For novel lncRNA prediction, 4 protein coding prediction software *PLEK* [35], *CPAT* [36], *CNCI* [37] and *CPC2* [38] were applied and 1184 novel transcripts showed non or low coding capability (Fig. 2b). Based on genomic located with nearby coding genes, we classified all novel lncRNAs into 5 categories: intronic lncRNA, sense lncRNA, antisense lncRNA, directional lncRNA and intergenic lncRNA, in which intronic lncRNAs accounted for 64.2% (Fig. 2c). Besides transcripts identification, alternative splicing events were analyzed. As shown in Fig. 2d, SE (Skipped exon) was the main alternative splicing type in all 5 types (SE-Skipped exon, A5SS-Alternative 5′ splice site, A3SS-Alternative 3′ splice site, MXE-Mutually exclusive exons and RI-Retained intron).

### DEGs and TTR-DEGs identification

**Fig. 3:**
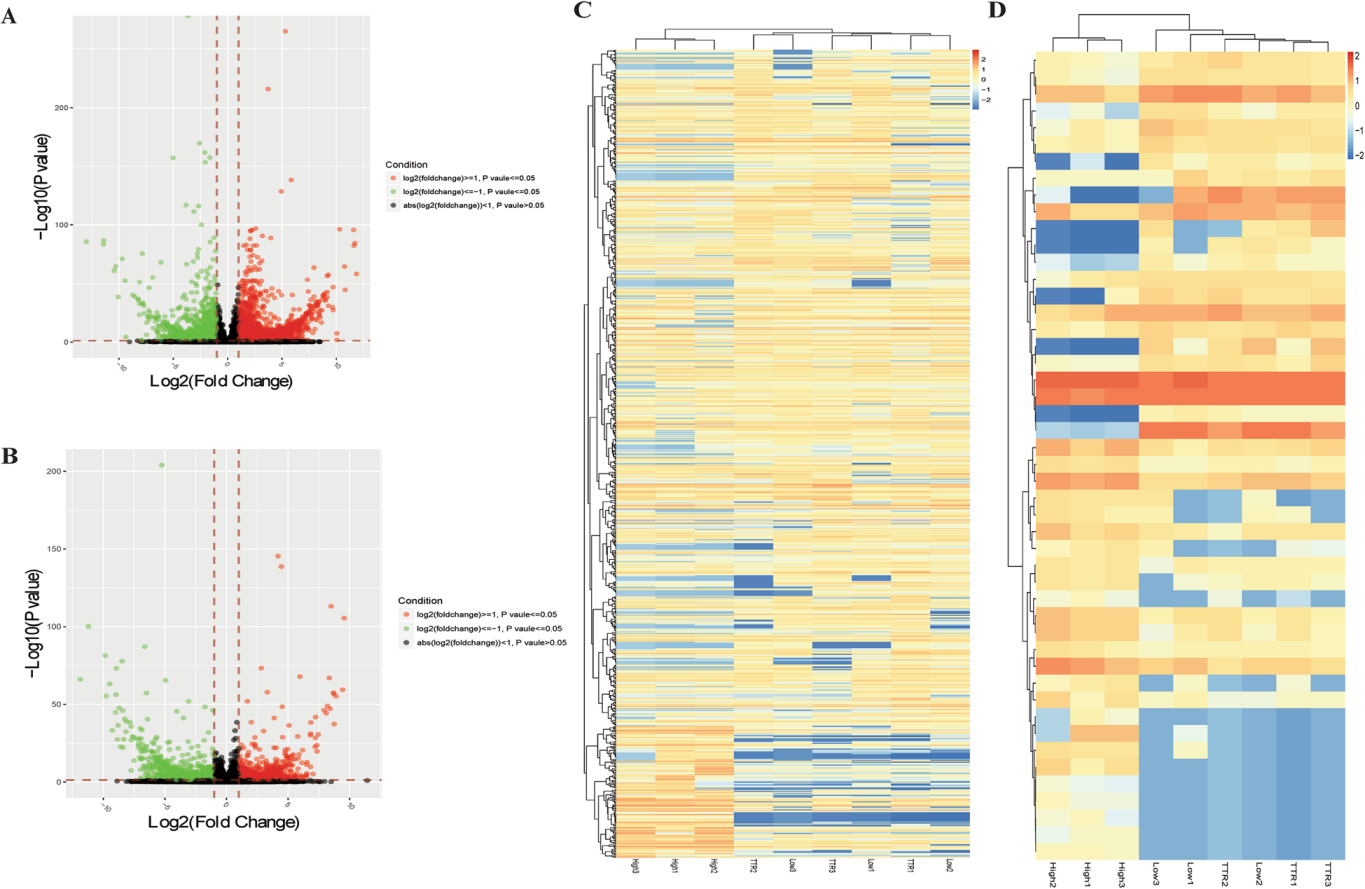
Significantly differential lncRNAs and mRNAs in glucose and TTR treated hRECs. The significantly DE-lncRNAs and DE-mRNAs were shown in volcano plot. **a** DE-lncRNAs and DE-mRNAs in HG group compared with LG group; **b** DE-lncRNAs and mRNAs in HG + TTR group compared with HG group. Significantly highly expressed genes (lncRNAs and mRNAs) were defined as genes with the value of fold change> = 2, *P* value<=0.05 and FDR < =0.05, were shown in red dots; significantly low expressed genes (lncRNAs and mRNAs) were defined as genes with the value of fold change<=0.5, *P* value<=0.05 and FDR < =0.05, and shown in green dots. Genes didn’t meet the criteria were shown in grey dots. After TTR-DGEs identification, hierarchical clustering and heatmap were used to show 853 mRNAs **c**) and 48 lncRNAs **d**) expression in 9 samples of 3 groups

Of all the lncRNAs and mRNAs identified, there were a total of 11,407 significantly differentially expressed mRNAs (DE-mRNAs) and 679 significantly differentially expressed lncRNAs (DE-lncRNAs) in HG groups, compared with LG group. Compared with HG group, there were 6206 significantly differentially expressed mRNAs (DE-mRNAs) and 194 significantly differentially expressed lncRNAs (DE-lncRNAs) in TTR + HG group. The DE-lncRNAs and DE-mRNAs were shown in volcano plot in Fig. 3a-b. As TTR exerts the protective effects comparing with high glucose, we mainly focused on DEGs with contradictory expression fold-change trend in TTR + HG group versus HG group and HG group versus LG group. A total of 853 mRNAs and 48 lncRNAs meet the criteria of opposite trend and were named as TTR related DEGs (TTR-DEGs), shown in Additional file 1: Table S1. Supervised hierarchical clustering was constructed to show TTR-DEGs expression in 9 samples of 3 groups (Fig. 3c-d). Samples in LG group and HG + TTR group were clustered together and showed similar expression pattern.

### KEGG pathway, gene ontology annotation and GSEA analysis

**Fig. 4:**
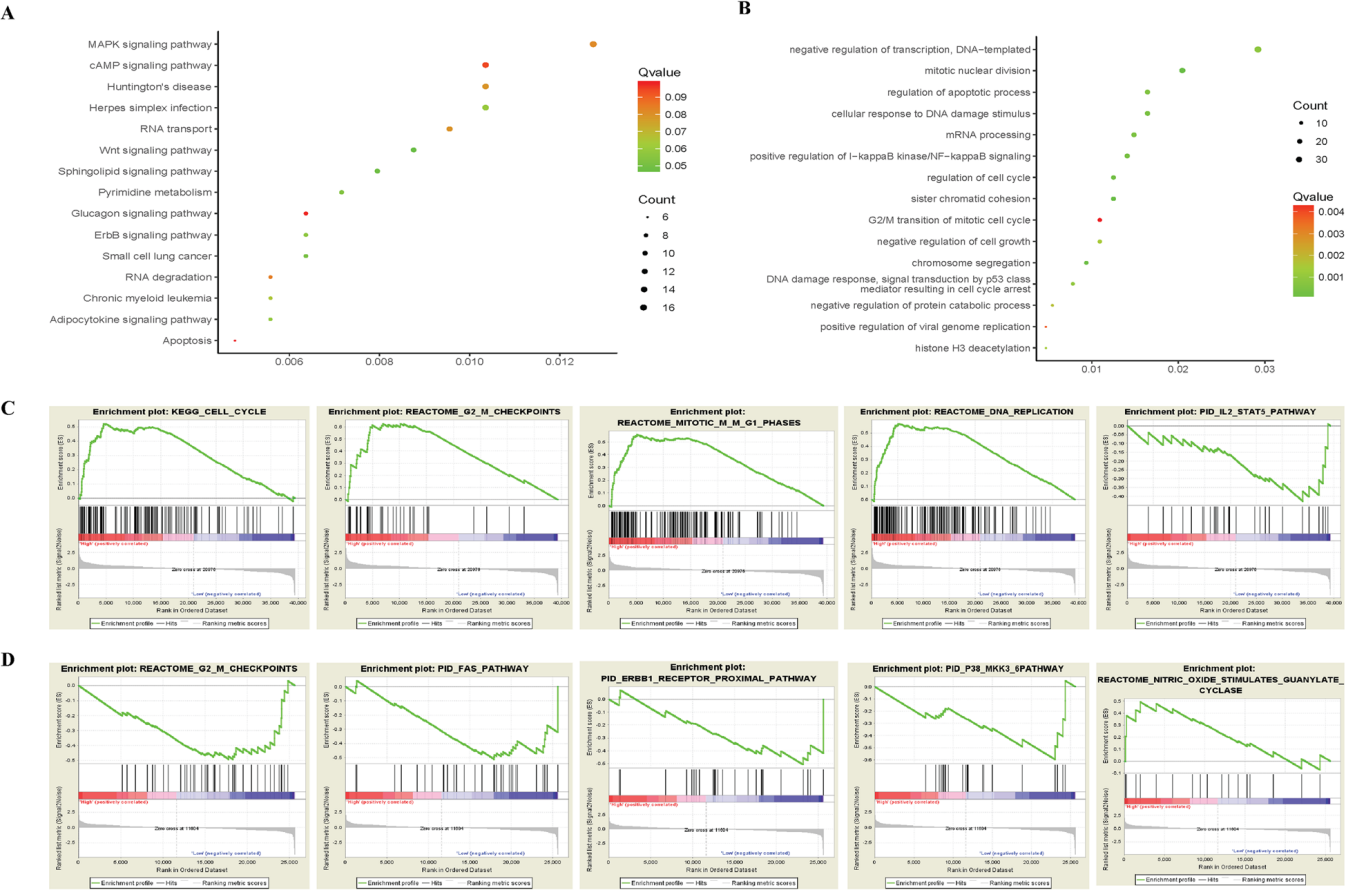
Function enrichment analyses of TTR-mRNAs and DE-mRNAs. KEGG pathway and GO-biological process (BP), cellular component (CC) and molecular function (MF) annotation and enrichment analysis and Gene Set Enrichment Analysis (GSEA) were used to study the function of 853 TTR-mRNAs. **a** TOP 15 significant enriched KEGG pathways like apoptosis, Glucagon signaling pathway, cAMP signaling pathway, MAPK signaling pathway, ErbB signaling pathway. **b** TOP 15 significant enriched GO biological processes like chromosome segregation, regulation of cell cycle, positive regulation of I-κB kinase/NF-κB signaling and cellular response to DNA damage stimulus. **c** GSEA analysis of DE-mRNAs in high glucose group compared with low glucose group identified Cell cycle related process like DNA replication and inflammatory pathways like IL2-STAT5. **d** GSEA analysis of DE-mRNAs in TTR group compared with high glucose group not only identified pathways involved in cell cycle like G2/M checkpoint, cell apoptosis pathways like Fas pathway, neovascularization like ERBB1/4 receptor pathway and oxidative stress response like Nitric Oxide Stimulates Guanylate Cyclase and P38-MKK3 pathway

Based on the premise that TTR exert the opposite role of high glucose, functional annotation and enrichment analysis were conducted to explore the role of 853 TTR-mRNAs. KEGG pathways analysis enriched pathways like apoptosis, glucagon signaling pathway, cAMP signaling pathway, RNA degradation and transport, MAPK signaling pathway, ErbB signaling pathway and Pyrimidine metabolism (Fig. 4a). The Gene Ontology analysis enriched biological processes like sister chromatid cohesion, chromosome segregation, regulation of cell cycle, positive regulation of I-κB kinase/NF-κB signaling and cellular response to DNA damage stimulus (Fig. 4b). In addition, all mRNA expression levels in LG, HG and HG + TTR groups were applied to conduct GSEA functional analysis. As shown in Fig. 3c, the GSEA analysis of mRNA in HG and LG identified Cell cycle related pathway like DNA replication, and inflammatory pathways like IL2-STAT5 (Fig. 4c). GSEA analysis of mRNA in TTR + HG and HG identified pathways involved in cell cycle like G2/M checkpoint, cell apoptosis pathways like Fas pathway, neovascularization like ERBB1/4 receptor pathway and oxidative stress response like Nitric Oxide Stimulates Guanylate Cyclase, and P38-MKK3 pathway (Fig. 4d).

### The TTR protein-protein interaction network and sub-networks function

**Fig. 5:**
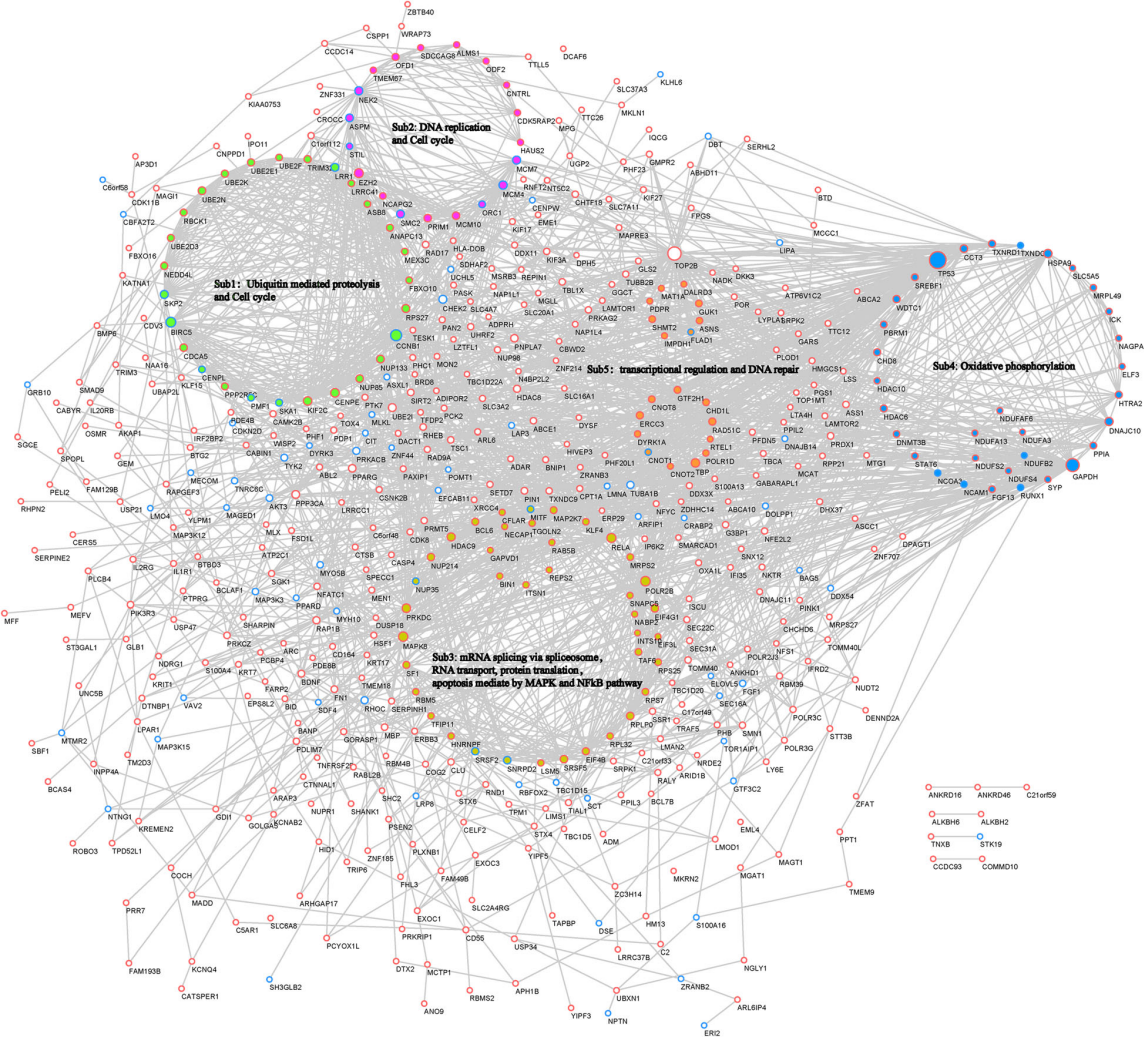
Protein-Protein interaction network and 5 identified sub-networks of TTR-mRNAs. The protein-protein interaction (PPI) network of 813 TTR-mRNAs was constructed with Cytoscape based on information in String database. Node size indicates surrounding gene numbers (degrees). Red node edge indicates TTR-mRNAs up-regulated in TTR groups (compared with HG group) and down-regulated in HG group (compared with LG group). Blue node edge indicates TTR-mRNAs with contrary trend. The MCODE application in Cytoscape identified 5 sub-networks. TTR-mRNAs in sub-network 1 were mainly involved in ubiquitin mediated proteolysis and cell cycle, with nodes filled in green color. TTR-mRNAs in sub-network 2 (nodes filled with purple color) were well known to influence DNA replication and cell cycle. Sub-network 3 TTR-mRNAs (nodes filled with brown color) were reported to regulate mRNA splicing via spliceosome, RNA transport, protein translation and apoptosis mediated by MAPK and NFκB pathway. Sub-network 4 TTR-mRNAs exert the role of Oxidative phosphorylation regulation and filled with blue color. TTR-mRNAs filled in orange color of Sub-network 5 participated in transcriptional regulation and DNA repair

To further elucidate the regulatory and interaction relationship, the 813 TTR-mRNAs were imported into the STRING database and visualized in Cytoscape. The STRING database identified a TTR-network of 548 TTR-mRNAs and 2135 edges (interaction relationship). Then the MCODE algorithm was implemented and the TTR-network was categorized into 5 sub-networks. The biological processes of TTR-mRNAs in each sub-network were analyzed. The 27 TTR-mRNAs in TTR sub-network 1 were mainly involved in ubiquitin mediated proteolysis and cell cycle. The 19 TTR-mRNAs in TTR sub-network 2 were well known to influence DNA replication and cell cycle. The 39 TTR-mRNAs in sub-network 3 were reported to regulate mRNA splicing via spliceosome, RNA transport, protein translation and apoptosis mediated by MAPK and NFκB pathway. The 32 TTR-mRNAs in sub-network 4 exert the role of Oxidative phosphorylation regulation. The function of 19 TTR-mRNAs in sub-network 5 was transcriptional regulation and DNA repair (Fig. 5).

### WGCNA analysis identified hub modules both regulated by glucose and TTR

**Fig. 6:**
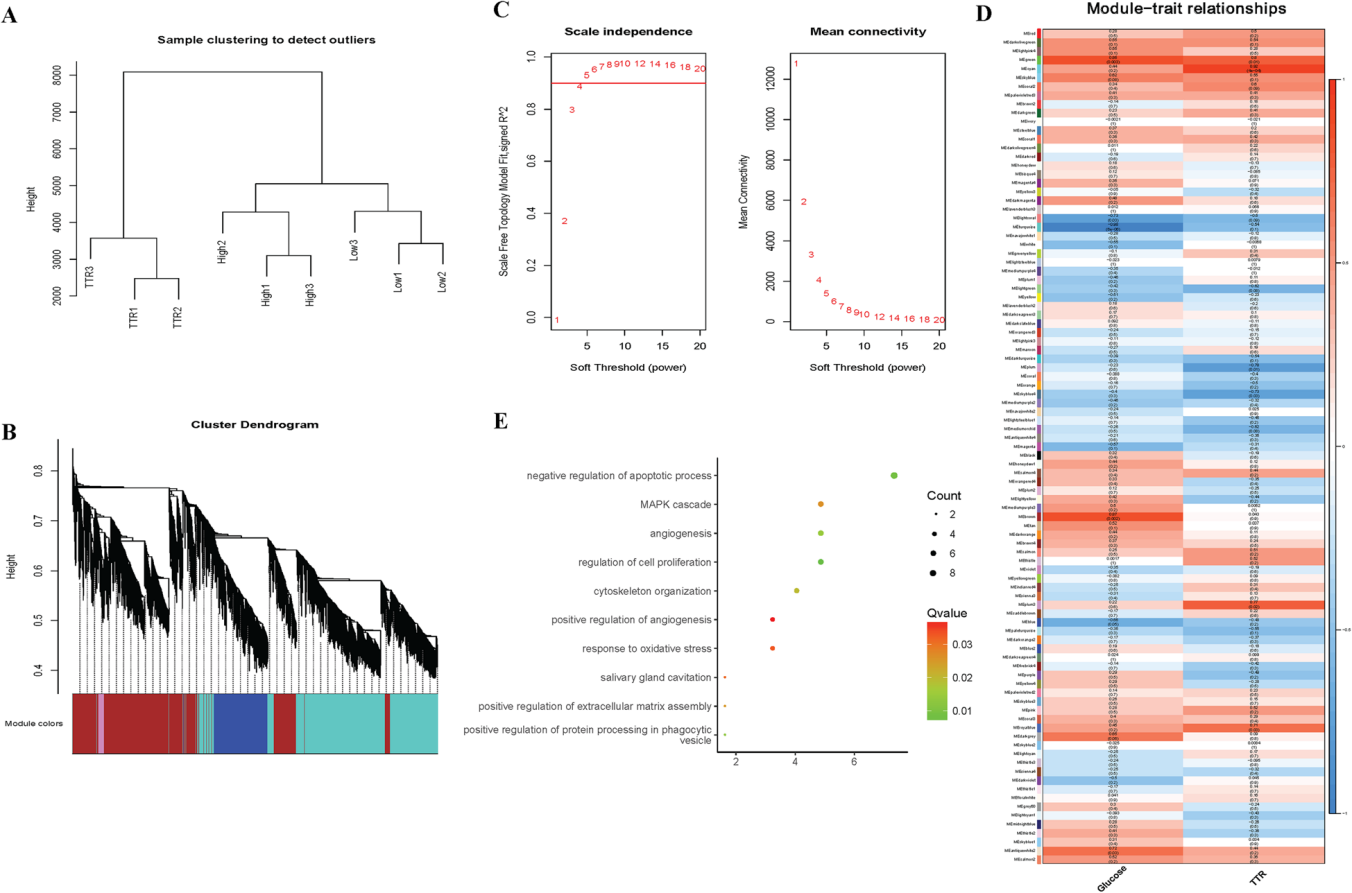
Weighted gene co-expression network analysis (WGCNA) analysis identified hub modules both regulated by glucose and TTR (**a**) Sample clustering as clustered to detect samples outliers and (**b**) cluster dendrogram was calculated; (**c**) Soft threshold calculation and mean connectivity to identify the soft threshold with best performance; (**d**) Gene expression module-trait (glucose and TTR) relationships were calculated when the soft threshold was 10; (**e**) the GO-biological processes of 133 genes in green module. The module genes were involved in biological processes like oxidative stress, angiogenesis, MAPK pathway, proliferation and apoptosis

**Fig. 7:**
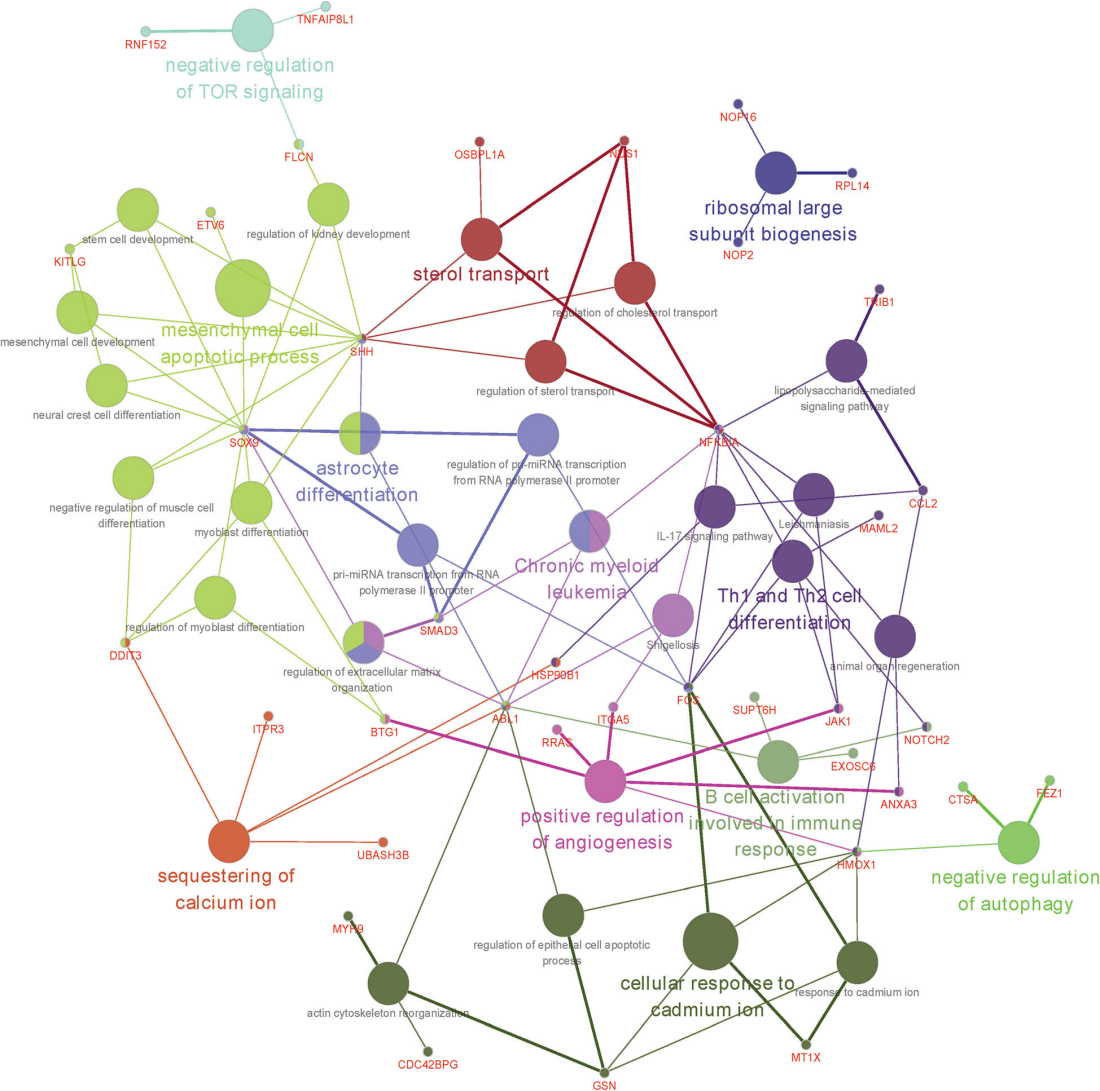
Gene-biological processes functional network of hub module. Functional analysis and enrichment of 133 genes in hub module was conducted with Cytoscape ClueGo application. Genes like *CTSA*, *FEZ1* and *HMOX1* were involved in biological processes like negative regulation of autophagy. Genes like *BTG1*, *ABL1*, *RRAS*, *ITGA5*, *JAK1*, *ANXA3* and *HMOX1* were reported to regulate angiogenesis

To further identify genes correlated with glucose and TTR treatment, gene expression module-traits (glucose and TTR) relationship were further illustrated with WGCNA analysis. As shown in Fig. 6a, sample clustering based on all gene expression characteristics generated 3 groups, conforming to known LG, HG and HG + TTR grouping. To further identify mRNAs both regulated by glucose and TTR, gene expression modules were calculated, of which module clusters were shown in Fig. 6b. Then the Soft threshold were calculated and set as 10 (Fig. 6c) and a hub module in MEgreen color showed largest Pearson correlation coefficient with both glucose and TTR (0.86 and 0.8, respectively, Fig. 6d). The hub module was comprised of 133 genes, and involved in biological processes like oxidative stress, angiogenesis, MAPK pathway, proliferation and apoptosis (Fig. 6e). The Cytoscape plug-in was used to annotate and visualize the gene-function network of 133 in the hub module. As shown in Fig. 7, genes like *CTSA* (Cathepsin A) [39], *FEZ1* (Fasciculation And Elongation Protein Zeta 1) [40] and *HMOX1* (Heme Oxygenase 1) [41] were involved in biological processes like negative regulation of autophagy. Genes like *BTG1 (BTG Anti-Proliferation Factor 1)* [42], *ABL1* (ABL Proto-Oncogene 1, Non-Receptor Tyrosine Kinase) [43], *RRAS* (RAS Related) [44], *ITGA5* (Integrin Subunit Alpha 5) [45], *JAK1* (Janus Kinase 1) [46], *ANXA3* (Annexin A3) [47] and *HMOX1* [48] were reported to regulate angiogenesis.

### LncRNA-mRNA regulatory network based on *cis*, *trans* and ceRNA interaction relationship

**Fig. 8:**
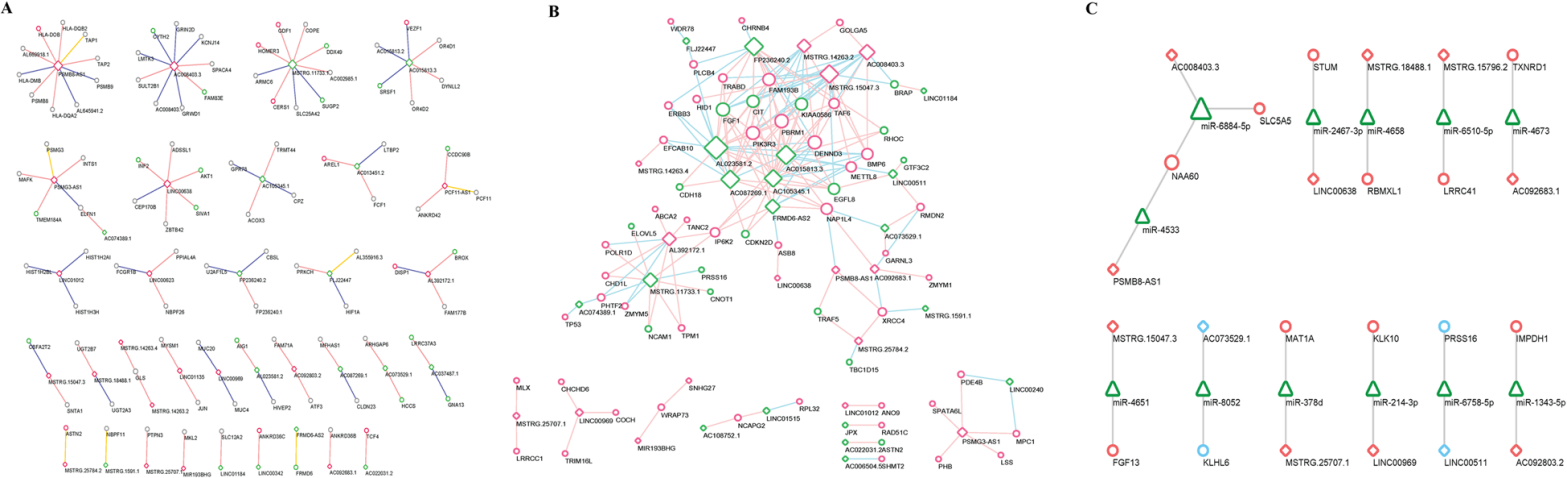
LncRNA-mRNA regulatory network based on *cis, trans* and ceRNA relationship. **a** The *cis* lncRNA-mRNA regulatory network. Up-regulated TTR-DEGs were shown in red color and down-regulated TTR-DEGs were shown in green color. Grey color indicates genes not significantly expressed. TTR-mRNAs were represented in circle nodes and TTR-lncRNAs were shown in diamond nodes. The size of nodes indicates degree of centrality.. Edges in the network denote relative upstream or downstream location within 10 kb in the same chromosome. Red edges indicate upstream, blue indicates downstream and yellow edges indicate overlapping location of lncRNAs compared with mRNA. **b** The *trans* lncRNA-mRNA regulatory network. TTR-mRNAs were represented in circle nodes and TTR-lncRNAs were shown in diamond nodes. Edges color in the network denotes positive or negative correlation between lncRNA and mRNA expression. Edges in red color indicated positive expression Pearson correlation and green edges denotes negative expression Pearson correlation. **c** The lncRNA-miRNA-mRNA network. LncRNAs were shown in diamond nodes, TTR-mRNAs in circle nodes and miRNAs in triangle

To identify lncRNA-mRNAs regulating network in glucose and TTR treated hRECs, lncRNA targets acting in *cis*, *trans* and ceRNA way was predicted. In *cis* regulatory network shown in Fig. 8a, lncRNAs nearby coding mRNAs were chosen for network construction. Edges in the network denote relative upstream or downstream location within 10 kb in the same chromosome. The *cis* regulatory network was composed of 33 *cis* lncRNA-mRNA pairs and 61 TTR-DEGs (33 TTR-lncRNAs and 28 TTR-mRNAs) genes of 133 genes. To further explore *trans*-regulatory network, Pearson correlation coefficient of gene expression were calculated and lncRNA-mRNA coefficient ≥ 0.95 or ≤ − 0.95 were screened. As shown in Fig. 8b, a network of 96 TTR-DEGs and 184 TTR-lncRNA/mRNA pairs was constructed in Cytoscape. In addition to *cis* and *trans* regulatory relationship, TTR-lncRNAs acting as ceRNAs of TTR-mRNA were predicted based on miRanda database. A total of 12 TTR-lncRNAs, 12 miRNAs and 12 mRNAs constituted 11 TTR-lncRNA/miRNA/mRNA sub-networks (Fig. 8c).

### The 5 functional lncRNA-mRNA *trans* networks

**Fig. 9:**
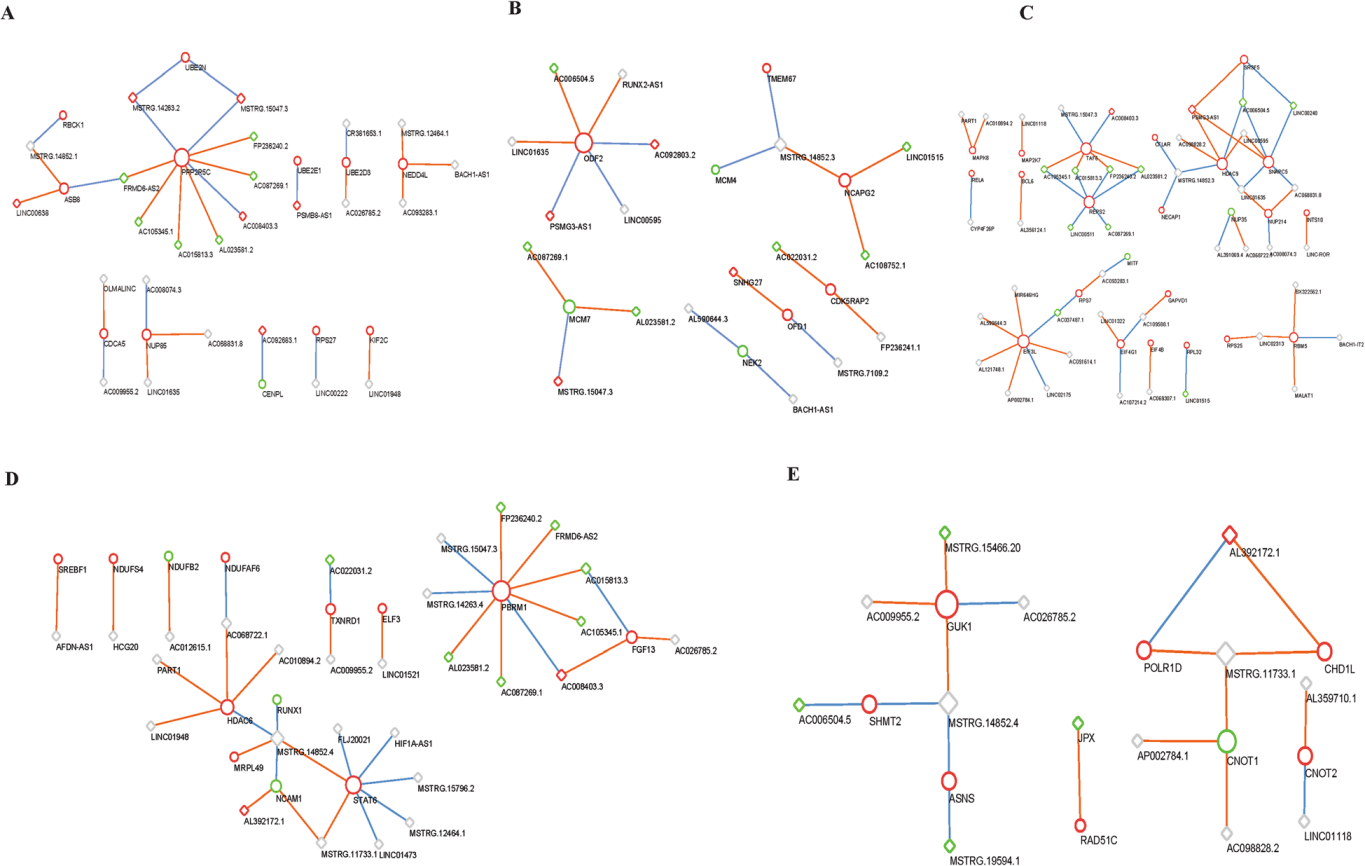
Five functional lncRNA-mRNA *trans* regulatory network. **a** TTR-lncRNAs-mRNA *trans* regulatory sub-network1: ubiquitin mediated proteolysis and cell cycle; **b** TTR-lncRNAs-mRNA trans regulatory sub-network2: DNA replication and cell cycle; **c** TTR-lncRNAs-mRNA trans regulatory sub-network3: mRNA splicing via spliceosome, RNA transport, protein translation and apoptosis mediated by MAPK and NFκB pathway; **d** TTR-lncRNAs-mRNA trans regulatory sub-network4: Oxidative phosphorylation regulation; **e** TTR-lncRNAs-mRNA *trans* regulatory sub-network5: transcriptional regulation and DNA repair

Integrating 5 mRNA sub-networks in Fig. 4 and lncRNA-mRNA *trans* network in Fig. 8b, we further constructed 5 functional lncRNA-mRNA networks. 12 TTR-lncRNAs correlated with 11 TTR-mRNAs in a potential *trans* sub-network 1 were involved in ubiquitin mediated proteolysis and cell cycle (Fig. 9a). 9 TTR-lncRNAs correlated with 7 TTR-mRNAs in a potential *trans* sub-network 2 were involved in DNA replication and cell cycle (Fig. 9b). 12 TTR-lncRNAs correlated with 23 TTR-mRNAs in a potential *trans* sub-network 3 were involved in mRNA splicing via spliceosome, RNA transport, protein translation and apoptosis mediated by MAPK and NFκB pathway (Fig. 9c). 9 TTR-lncRNAs correlated with 12 TTR-mRNAs in a potential *trans* sub-network 4 were involved in Oxidative phosphorylation regulation (Fig. 9d). 5 TTR-lncRNAs correlated with 8 TTR-mRNAs in a potential *trans* sub-network 5 were involved in transcriptional regulation and DNA repair (Fig. 9e).

### A 3-lncRNA centered TTR regulatory network with diagnostic performance

**Fig. 10:**
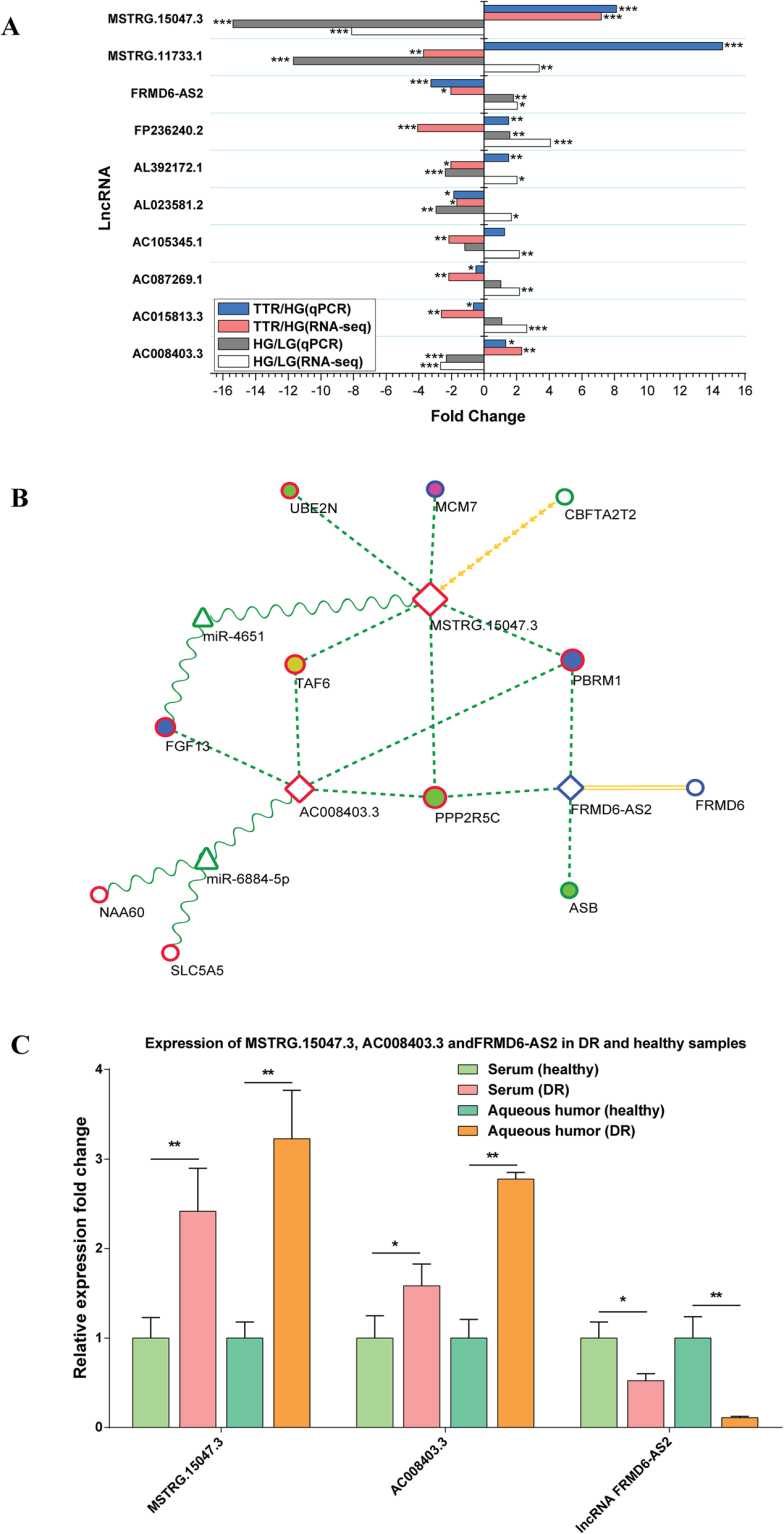
LncRNA expression validation and 3-lncRNA centered TTR regulatory network. **a** The 10 TTR-lncRNAs expression fold change detected with RNA-Seq and qRT-PCR, respectively. * *P* < 0.05, ** *P* < 0.01, *** *P* < 0.001. **b** A 3-lncRNA centered TTR regulatory network. 3 TTR-lncRNAs were shown in diamond nodes, TTR-mRNAs in circle nodes and miRNAs in triangle. Equal dash edges indicate *trans* relationship. Yellow separate arrow pointing from *CBFTA2T2* to *MSTRG.15047.3* indicates upstream nearby location. Yellow parallel edges between FRMD6-AS2 and FRMD6 represent overlapping and antisense location. Sinewave edges between TTR-lncRNAs, TTR-mRNAs with miRNAs denotes potential ceRNA relationship. TTR-mRNAs filled in green (*PPP2R5C*, *UBE2N*and *ASB*), purple (*MCM7*), brown (*TAF6*) and blue (*FGF13* and *PBRM1*) indicates genes in sub-network 1–4. **c** The expression of lncRNA *MSTRG.15047.3*, *AC008403.3* and *FRMD6-AS2* were examined with qRT-PCR in aqueous humor and serum samples from 30 DR patients and 10 healthy participants. * *P* < 0.05, ** *P* < 0.01, *** *P* < 0.001

To further pinpoint the regulatory and functional network centered by lncRNAs in TTR and glucose on hRECs, the expression of 10 TTR-lncRNAs with the largest node size (indicating largest number of direct surrounding genes in the *trans*-network), *AL023581.2*, *AL392172.1*, *FP236240.2*, *AC105345.1*, *AC087269.1*, *AC015813.3*, *FRMD6-AS2*, *AC008403.3*, *MSTRG.15047.3* and *MSTRG.11733.1* were validated by qRT-PCR in hRECs. As shown in Fig. 10a, 3 TTR-lncRNAs (*MSTRG.15047.3*, *FRMD6-AS2* and *AC008403.3*) showed concordant significant expression trend in both RNA-Seq and qRT-PCR detection methods. The predicted novel lncRNA *MSTRG.15047.3* in LG and HG + TTR groups was significantly up-regulated by 15.39 fold and 8.11 fold, compared with HG groups, respectively. LncRNA *FRMD6-AS2* (FERM domain-containing protein 6 Antisense RNA 2) in LG and HG + TTR groups showed decreased expression of 1.81 and 3.25 fold, compared with HG group, respectively. LncRNA *AC008403.3* in LG and HG + TTR groups was significantly up-regulated with a fold change of 2.32 and 1.33, compared with HG group, respectively. Then we focused on the 3 TTR-lncRNAs and integrated predicted targets in *cis, trans* and ceRNA way, and built a 3-lncRNA centered TTR regulatory network (Fig. 10b). Finally, the expression of lncRNA *MSTRG.15047.3, AC008403.3* and *FRMD6-AS2* was examined in aqueous humor and serum samples from 30 DR patients and 10 normal controls (patients without diabetes). In DR patients, *MSTRG.15047.3* and *AC008403.3* showed significantly relative higher expression in both aqueous humor and serum samples, compared with normal controls, and *FRMD6-AS2* was significantly down-regulated (Fig. 10c), showing promising potential as DR diagnostic biomarkers.

## Discussion

Recently, it was reported that dysfunction of TTR was bound up with system diseases and the intervention of TTR improved multiple clinical manifestations of hereditary transthyretin amyloidosis [49, 50]. TTR was also involved in various biological processes such as proteolysis, nerve regeneration, autophagy, glucose homeostasis and angiogenesis regulation [51]. In ophthalmic diseases, abnormal TTR expression was reported in high myopia and diabetic retinopathy patients. Exogenous TTR treatment inhibited hRECs proliferation, migration and tube forming capability and induced apoptosis in vitro [12, 13]. However, the underlying mechanism still remains unknown. In this study, through RNA-sequencing, the basic transcriptome characteristics of hRECs underlying hyperglycemia and TTR with high glucose conditions were first determined. Then, through functional annotation, protein-protein interaction and co-expression analysis, the protective role of TTR in hRECs dysfunction including signaling pathways, biological processes and trait modules were further explored. Furthermore, potential targets of TTR related lncRNAs were predicted and 3 coding-non-coding networks were constructed based on putative lncRNA acting mode. Finally, a 3-lncRNA centered regulatory hub network with clinical significance was proposed to explain the function of TTR attenuating neovascularization.

The functional annotation of TTR related mRNAs corroborated previous reports of relative decreased expression of TTR in DR patients and neovascularization function. In our study of TTR function in hRECs, biological processes involved in cell proliferation, migration and neovascularization were significantly enriched, in lining with studies that TTR modulated human umbilical vein endothelial cells (HUVECs) apoptosis and inhibit migration, regulation of hRECs apoptosis, proliferation and migration. In addition, biological processes and pathways like response to oxidative stress, inflammatory signaling like IL2-STAT5, and regulation of autophagy were also enriched and reported for the first time. The intricate cross-talking between oxidative stress, inflammatory signaling pathway, autophagy and apoptosis were reported in melatonin ameliorating DR progression [52]. Thus, we speculated that there may exist a similar regulatory network, composed of oxidative stress, inflammation signaling, autophagy and apoptosis, linking the protective function of TTR and DR. The 5 protein-protein interaction sub-networks in Fig. 4 implied that TTR might orchestrate pathways in transcriptional and post-transcriptional levels. Genes in sub-network 1 implied that TTR might regulate cell cycle via ubiquitin mediated proteolysis, and the function of sub-network 3 suggested that TTR modulates MAPK and NF-κB pathway through RNA transportation, splicing and translation, post-transcriptionally. In transcriptional level, the function of genes in the sub-network 5 was focused on DNA repair, an important link of oxidative stress, inflammatory signaling, autophagy and apoptosis [53–55]. Hence, the functional annotation provides a comprehensive direction for further research of how TTR rescuing high glucose induced neovascularization abnormality.

Another important part of our work was the investigation of TTR related lncRNAs and their potential regulatory role in DR. As non-coding RNAs, the functional mode of lncRNAs via regulating mRNA expression in *cis*, *trans* or as miRNA sponge has been well recognized in angiogenesis and vascular diseases [56]. LncRNA *MEG3* knockdown aggravated retinal vessel dysfunction by the activation of PI3k/Akt signaling [16]. Through regulating p38-MAPK pathway, *HOTTIP* improved diabetic retinal microangiopathy [19]. As an important inflammation regulator, lncRNA *MALAT1* was reported to affect retinal endothelial cell proliferation, migration, and tube formation through cross-talking with p38 MAPK signaling pathway [20, 21]. As *miR-29b* and *miR-150-5p* sponge or *VEGF* ceRNA, lncRNA *MIAT* (Myocardial infarction associated transcript) was reported to regulate endothelial cell apoptosis [18]. Another role that well studied in cardiovascular diseases is lncRNA *ANRIL* (antisense non-coding RNA in the INK4 locus), which regulated *VEGF* expression and function in diabetic retinopathy via *miR-200b*, and *EZH2* (Enhancer of Zeste 2 Polycomb Repressive Complex 2 Subunit) of the *PRC2* (Polycomb Repressive Complex 2) complex [23]. However, the role of lncRNAs in TTR ameliorating hRECs dysfunction has not yet been reported. In this paper, a 3 lncRNA-mRNAs regulatory network and 5 lncRNAs-mRNAs sub-networks in *cis*, *trans* and ceRNA were constructed for the first time. The networks would contribute to understanding the underlying mechanism of TTR functions in DR from the perspective of different lncRNAs acting mode.

In addition, expression of 10 lncRNAs in lncRNA-mRNAs networks were validated by qRT-PCR and 3 lncRNAs including 2 up-regulated novel lncRNAs, i.e., *MSTRG.15047.3* and *AC008403.3*, 1 down-regulated lncRNA, i.e., *FRMD6-AS2* (FRMD6 Antisense RNA 2). The 3 lncRNAs formed a complicated potential interacting network, including 11 mRNAs and 2 miRNAs. Based on the network, the novel predicted lncRNA *MSTRG.15047.3* might interact with nearby up-located *CBFTA2T2* in a *cis*-regulatory way, *trans*-regulates cell cycle and neovascularization via *UBE2N* (Ubiquitin Conjugating Enzyme E2 N), *PPP2R5C* (Protein Phosphatase 2 Regulatory Subunit B′ Gamma), *MCM7*(Minichromosome Maintenance Complex Component 7), *TAF6* (The TATA-Box Binding Protein Associated Factor 6) and *PBRM1* (Polybromo 1), or just sponge *miR-4651* and promote *FGF13* (Fibroblast Growth Factor 13) expression. Gene *UBE2N* encoded a member of the E2 ubiquitin-conjugating enzyme family and was reported to be involved in cell cycle and error-free DNA repair pathway [57]. The *UBE2V1* (Ubiquitin Conjugating Enzyme E2 V1)*-UBE2N* heterodimer acts in concert with *TRIM5* (Tripartite Motif Containing 5) to activate the MAP 3 K7/TAK1 complex and induce expression of NFκB and MAPK-responsive inflammatory genes [58, 59]. *PPP2R5C* gene belongs to the phosphatase 2A regulatory subunit B family. The *PP2A* (Protein Phosphatase 2 Phosphatase Activator) *-PPP2R5C* holoenzyme may specifically dephosphorylate and activate *TP53* (Tumor Protein P53) and play a role in DNA damage-induced inhibition of cell proliferation, as well as regulate the ERK signaling pathway through ERK dephosphorization [60]. *MCM7* acts as component of the MCM2–7 complex (MCM complex) and may be involved in the formation of replication forks and in the recruitment of other DNA replication related proteins [61]. The TATA-Box Binding Protein Associated Factor 6 (*TAF6*) are components of the transcription factor IID (TFIID) complex and modulates cell apoptosis in a *TP53* dependent [62] and independent [63] way. *PBRM1* encodes a subunit of ATP-dependent chromatin-remodeling complexes. In clear cell renal cancer, concomitant loss of *PBRM1* rescues VHL-induced replication stress, maintaining cellular fitness and allowing proliferation [64]. Fibroblast Growth Factor 13 possesses broad mitogenic and cell survival activities, including embryonic development, cell growth, morphogenesis and invasion [65]. For *AC008403.3*, in addition to sharing 4 *trans* targets of *TAF6*, *PPP2R5C*, *PBRM1* and *FGF13* with *MSTRG.15047.3* may act as a ceRNA of *NAA60* (N (Alpha)-Acetyltransferase 60, NatF Catalytic Subunit) and *SLC5A5* (Solute Carrier Family 5 Member 5) via sponging *miR-6884-5p*. *NAA60* encodes an enzyme that localizes to the Golgi apparatus and transfers an acetyl group to the N-terminus of free proteins [66]. *SLC5A5* encodes a member of the sodium glucose cotransporter involved in thyroxine metabolism, a shared molecular function of TTR [67]. As the antisense RNA and potential cis-regulator of *FRMD6* (FERM Domain Containing 6), a known hippo pathway kinase regulator [68], lncRNA *FRMD6-AS2* might interact with *PBRM1*, *PPP2R5C* and *ASB* (Arylsulfatase B) to regulate cell proliferation and neovascularization. Further studies will be conducted to explore the exact role of 3-lncRNA regulatory network linking TTR and DR neovascularization.

Finally, lncRNAs *MSTRG.15047.3*, *AC008403.3* and *FRMD6-AS2* showed significant differential expression levels in aqueous humor and serum samples of DR patients and normal controls (patients without diabetes), made possible 3 lncRNAs as diagnostic biomarkers in DR. Several studies investigated the clinical significance of lncRNAs in DR, like *MIAT* [17] and *MALAT1* [21]. In this study, we proposed 3 of 10 lncRNAs potentially involved in DR progression, and we would explore their function and molecular mechanism in the future. Additionally, the correlation of lncRNAs expression with clinical parameters, and especially performance as DR biomarkers would be studied, with more samples, including healthy donors and other ocular diseases.

In summary, it was concluded that TTR repressed hRECs neovascularization, via a 3-lncRNA regulatory network. This investigation was mainly based on glucose and exogenous TTR treatment of hRECs, RNA sequencing, bioinformatics analysis and qRT-PCR validation in clinical samples. There are several limitations in this study. First, hRECs cell line was used as the DR cell model, and the tube forming assay demonstrated that TTR repressed neovascularization. Whether the neovascularization repressing effect of TTR and 3-lncRNA regulatory network were restricted to hRECs, or represents a general mechanism in microvascular endothelial cells is an important question. In fact, Kun Shan .et al. employed other microvascular endothelial cell lines, like human umbilical vein endothelial cells (HUVECs), EA.hy.926 and human coronary artery endothelial cells (HCAECs) to investigate retinal vascular dysfunction [69]. In addition, using primary human retinal microvascular endothelial cell (HRMECs) would help to illustrate the point. Secondly, this study was conducted in vitro, and investigation of TTR effect in DR animal models, like streptozotocin (STZ) administration, pancreatectomy and genetic models [70] would provide more solid evidence to support the conclusion. Thirdly, an exogenous TTR was employed to examine the function and a vector or virus mediated TTR overexpression approach would provide more details about the endogenous function of TTR. Lastly, we speculated that the 3-lncRNA regulatory network play a pivotal role in TTR function, and more experiments, like rescue assays of 3 lncRNAs knockdown or overexpression in TTR treatment cells, would help to support the point.

## Conclusion

In summary, we investigated (1) the basic characteristics and landscape of RNA transcriptome in TTR and glucose treated hRECs; (2) the function of TTR-mRNAs from signaling pathway, biological process, subnetworks and hub modules; (3) lncRNA-targets networks and functional sub-networks in *cis*, *trans* and ceRNA regulatory way; (4) a 3-lncRNA centered hub network that might mediate the comprehensive function of TTR and serve as potential diagnostic biomarkers in DR. The research would pave the way for understanding the complex biology of TTR in DR progression.

## Supplementary information

**Additional file 1:** **Table S1.** We listed the TTR-mRNAs and TTR-lncRNAs, respectively. A total of 853 TTR-mRNAs/transcripts and 48 TTR-mRNAs/transcripts with normalized expression level in low glucose (LG), high glucose (HG) and TTR + TTR co-treated (TTR) groups, relative expression fold change (log2 transformed) with *P* value and FDR in TTR/HG and HG/LG groups, gene ID and gene symbol were shown.

**Additional file 2: Figure S1.** Transcript specific primers targeting lncRNAs were designed. As LncRNA AC008403.3 was co-localized with LMTK3 in chromosome 19 and FRMD6-AS2 and FRMD6 were localized in DNA+/− strands, with partial sequences overlapping (Figure S1), transcript specific primers were designed.

**Additional file 3: Figure S2.** HRECs cellular morphology in different treatment conditions. HRECs cellular morphology and physiological nature didn’t vary under different treatment conditions as the treatment is for a long time of 48 h.

## Data Availability

Transcriptome data have been submitted to GEO repository: GSE117238.

## Abbreviations

ABL1: ABL Proto-Oncogene 1, Non-Receptor Tyrosine Kinase
ACTB: Beta-Actin
Angpt2: Angiopoietin 2
ANRIL: Antisense non-coding RNA in the INK4 locus
ANXA3: Annexin A3
ASB: Arylsulfatase B
BDNF-AS: BDNF Antisense RNA
BTG1: BTG Anti-Proliferation Factor 1
CeRNA: Competing endogenous RNA
CNC nework: Coding-non-coding network
CNCI: Coding-Non-Coding Index
CPAT: Coding Potential Assessing Tool
CPC2: Coding Potential Calculator 2
CTSA: Cathepsin A
DAVID: The Database for Annotation, Visualization and Integrated Discovery
DEGs: Differentially expressed genes
DE-lncRNAs: Differentially expressed long non-coding RNAs
DE-mRNAs: Differentially expressed mRNAs
DR: Diabetic retinopathy
EZH2: Enhancer of Zeste 2 Polycomb Repressive Complex 2 Subunit
FDR: False discovering rate
FEZ1: Fasciculation And Elongation Protein Zeta 1
FGF13: Fibroblast Growth Factor 13
FRMD6-AS2: FRMD6 Antisense RNA 2
GAPDH: Glyceraldehyde-3-phosphate dehydrogenase
GO: Gene Ontology
GRP78: Glucose-regulated protein 78
GSEA: Gene Set Enrichment Analysis
HBMEC-2: Human brain microvascular endothelial cell
HCAECs: Human coronary artery endothelial cells
HG + TTR: High glucose with 4 μM TTR
HG: High glucose
HMOX1: Heme Oxygenase 1
HOTTIP: HOXA Distal Transcript Antisense RNA
HRECs: Human retinal endothelial cells
HRMECs: Human retinal microvascular endothelial cells
HRPECs: Human retinal pigment epithelial cells
ITGA5: Integrin Subunit Alpha 5
JAK1: Janus Kinase 1
KEGG: Kyoto Encyclopedia of Genes and Genomes
LG: Low glucose
LncRNAs: Long non-coding RNAs
MALAT1: Metastasis Associated Lung Adenocarcinoma Transcript 1
MCM7: Minichromosome Maintenance Complex Component 7
MCODE: Molecular COmplex Detection
MEG3: Maternally Expressed 3
MIAT: Myocardial Infarction Associated Transcript
MRE: miRNA response element
NAA60: N (Alpha)-Acetyltransferase 60, NatF Catalytic Subunit
PBRM1: Polybromo 1
PLEK: Predictor of long non-coding RNAs and messenger RNAs based on an improved k-mer scheme
PP2A: Protein Phosphatase 2 Phosphatase Activator
PPI: protein-protein interaction
PRC2: Polycomb Repressive Complex 2
RBP: Retinol-binding protein
RRAS: RAS Related
SLC5A5: Solute Carrier Family 5 Member 5
Sox2-OT: SOX2 Overlapping Transcript
STR: Short Tandem Repeat
STZ: Streptozotocin
TAF6: The TATA-Box Binding Protein Associated Factor 6
TFIID: Transcription factor IID
TP53: Tumor Protein P53
TRIM5: Tripartite Motif Containing 5
TTR: Transthyretin
TTR-DEGs: TTR related DEGs
UBE2V1: Ubiquitin Conjugating Enzyme E2 V1
VEGF2: Vascular Endothelial Growth Factor
VEGFR1: Vascular Endothelial Growth Factor Receptor 1
WGCNA: Weighted gene co-expression network analysis
