# Supplementary figures and images for: Transcriptome analysis identified a novel 3-LncRNA regulatory network of transthyretin attenuating glucose induced hRECs dysfunction in diabetic retinopathy
Source: BMC Med Genomics. 2019 Oct 15;12:134. doi: 10.1186/s12920-019-0596-2 (PMC6794807; doi:10.1186/s12920-019-0596-2)

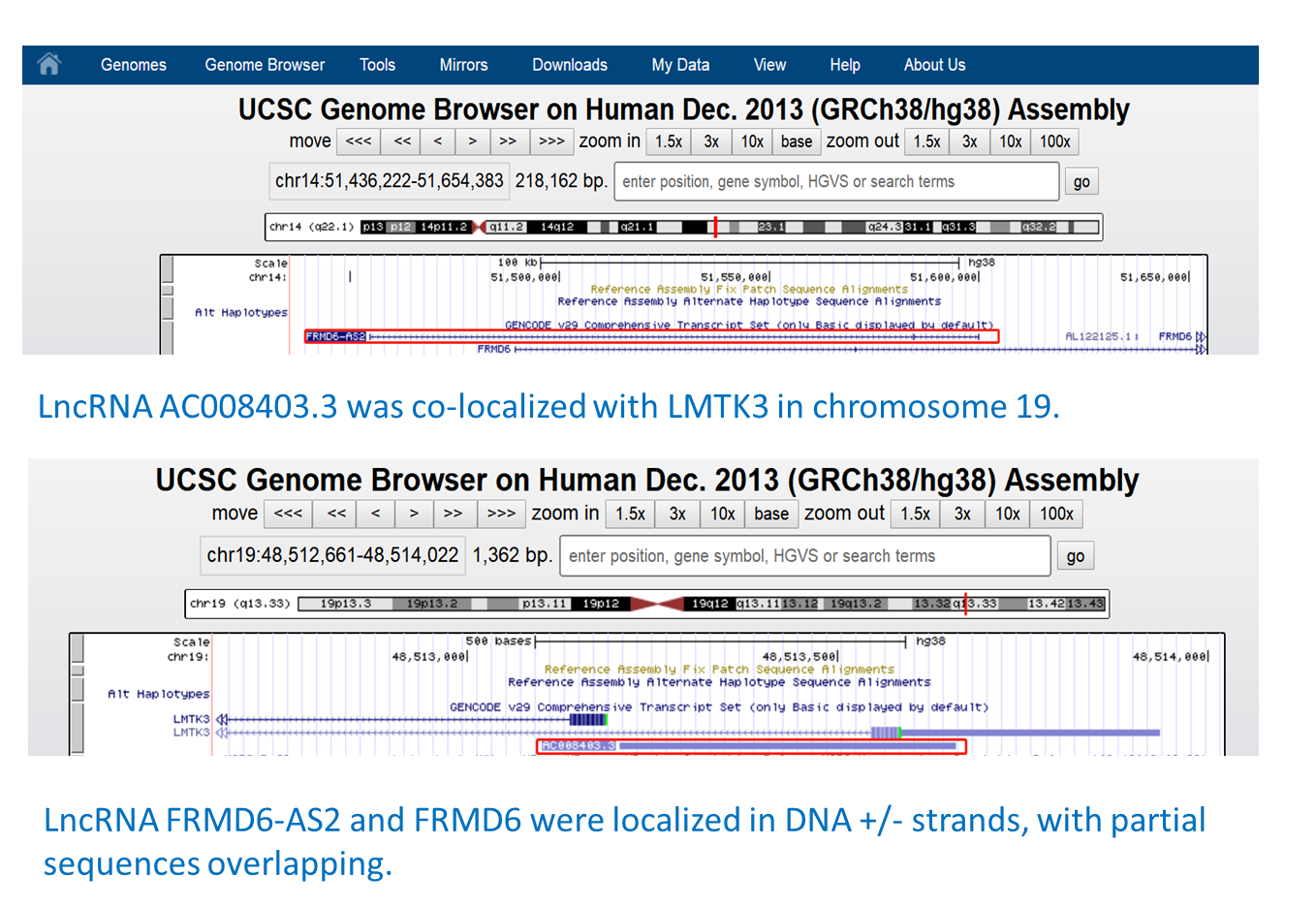

Supplement: Supplementary file 2 — Additional file 2: Figure S1. Transcript specific primers targeting lncRNAs were designed. As LncRNA AC008403.3 was co-localized with LMTK3 in chromosome 19 and FRMD6-AS2 and FRMD6 were localized in DNA+/− strands, with partial sequences overlapping (Figure S1), transcript specific primers were designed. [file 12920_2019_596_MOESM2_ESM.tif]

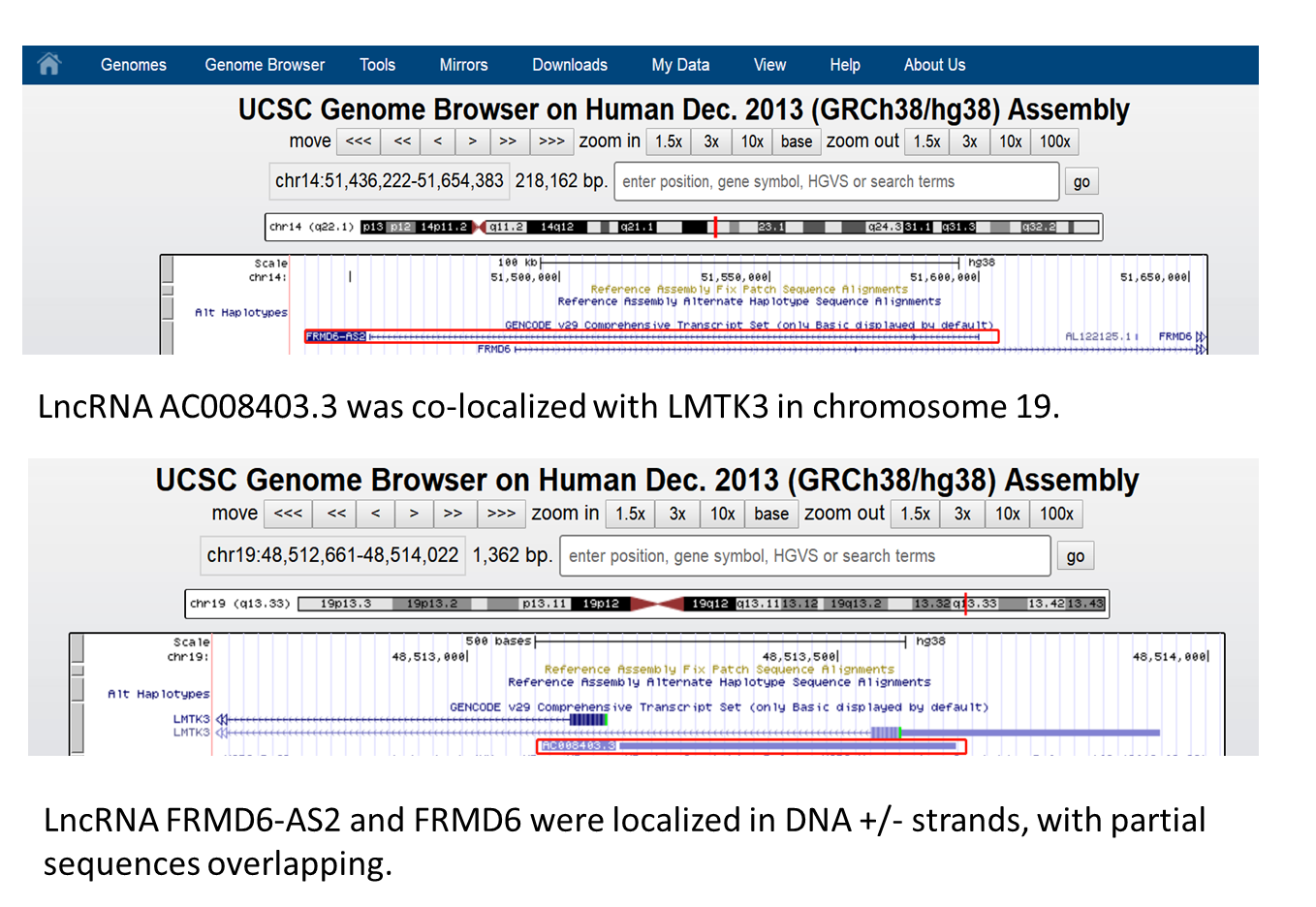

Supplement: Supplementary file 3 — Additional file 3: Figure S2. HRECs cellular morphology in different treatment conditions. HRECs cellular morphology and physiological nature didn’t vary under different treatment conditions as the treatment is for a long time of 48 h. [file 12920_2019_596_MOESM3_ESM.tif]
